# Melatonin activates ABCA1 via the BiP/NRF1 pathway to suppress high-cholesterol-induced apoptosis of mesenchymal stem cells

**DOI:** 10.1186/s13287-021-02181-4

**Published:** 2021-02-05

**Authors:** Jun Sung Kim, Young Hyun Jung, Hyun Jik Lee, Chang Woo Chae, Gee Euhn Choi, Jae Ryong Lim, Seo Yihl Kim, Joo Eun Lee, Ho Jae Han

**Affiliations:** 1 Department of Veterinary Physiology, College of Veterinary Medicine, Research Institute for Veterinary Science, and BK21 Four Future Veterinary Medicine Leading Education & Research Center, Seoul National University, Seoul, 08826 Republic of Korea; 2 Laboratory of Veterinary Physiology, College of Veterinary Medicine, Chungbuk National University, Cheongju, Chungbuk 28644 Republic of Korea; 3 Institute for Stem Cell & Regenerative Medicine (ISCRM), Chungbuk National University, Cheongju, Chungbuk 28644 Republic of Korea

**Keywords:** Mesenchymal stem cells, Cholesterol efflux, Nuclear factor erythroid 2-related factor 1, Stem cell transplantation, Obese mouse wound healing

## Abstract

**Background:**

Retarded wound healing in patients with obesity contributes to a risk of complications associated with vascular insufficiency and oxidative stress. The high cholesterol levels of patients with obesity are associated with apoptosis of engrafted umbilical cord blood-derived mesenchymal stem cells (UCB-MSCs). Melatonin contributes to the prevention of cholesterol accumulation in patients with obesity via a mechanism that is poorly understood. We therefore investigated the regulatory mechanism of melatonin in cholesterol-induced apoptosis.

**Methods:**

The protective effects of melatonin on cholesterol-induced apoptosis were investigated in UCB-MSCs. We used a mouse model of induced obesity to show that melatonin treatment restored the survival rate of transplanted UCB-MSCs and their wound-healing capacity. The mean values of the treatment groups were compared with those of the control group using Student’s *t* test, and differences among three or more groups were analyzed using one-way analysis of variance with Dunnett’s multiple comparison test.

**Results:**

Melatonin treatment increased the expression of ATP-binding cassette subfamily A member 1 (ABCA1), which reduced cholesterol accumulation and cholesterol-induced apoptosis. The mouse skin wound healing model showed that melatonin treatment restored the survival rate of transplanted UCB-MSCs and the wound-healing capacity of obese mice. Melatonin inhibited the expression of binding immunoglobulin protein (BiP) through the regulation of MT2/Sp1-dependent microRNA-597-5p. Melatonin decreased the co-localization of BiP with nuclear factor erythroid 2-related factor 1 (NRF1), which resulted in increased ABCA1 expression.

**Conclusion:**

Melatonin induced the efflux of intracellular cholesterol through ABCA1 to decrease apoptosis of UCB-MSCs via an MT2-dependent BiP/NRF1 pathway.

**Supplementary Information:**

The online version contains supplementary material available at 10.1186/s13287-021-02181-4.

## Background

The endogenous indoleamine hormone melatonin (*N*-acetyl-5-methoxytryptamine) is synthesized and secreted by many organs, including the pineal gland, which regulates the day/night sleep cycle [1]. Melatonin regulates physiological processes in cells, including regulation of oxidative stress, cell migration, and apoptosis [2]. Moreover, melatonin and cholesterol levels are associated with obesity [3], and melatonin decreases cholesterol absorption and accumulation in rats [4]. Furthermore, melatonin inhibits the activation of sterol regulatory element-binding transcription factor 1c, fatty acid synthase, and stearoyl-CoA desaturase 1-associated lipogenesis and increases peroxisome proliferator-activated receptor-α-dependent lipolysis [5, 6]. However, the mechanism by which melatonin regulates the efflux of intracellular cholesterol is unknown.

Nuclear factor erythroid 2-related factor 1 (NRF1, also known as NFE2L1) is required to maintain cholesterol homeostasis via repression of ATP-binding cassette subfamily A member 1 (ABCA1) expression [7]. NRF1, a member of the cap‘n’colar basic leucine zipper family, is present on the endoplasmic reticulum (ER) membrane, and the nuclear translocation of NRF1 is stringently regulated by HMG-CoA reductase degradation 1 (HRD1)-dependent ER-associated degradation (ERAD) [8]. The cholesterol recognition/interaction amino acid consensus sequence domain of NRF1 directly binds to cholesterol in the ER membrane, which influences the suppression of HRD1-dependent ERAD and nuclear translocation of NRF1 [7]. The substrate of ERAD is recognized by proteins such as binding immunoglobulin protein (BiP), OS9 ER lectin, and store-operated calcium entry-associated regulatory factors. These proteins bind to protein sel-1 homolog 1, the core of the HRD1 complex, to transfer the substrate to HRD1 [9]. Elevated cholesterol levels lead to downregulation of NRF1 ubiquitination and degradation through ERAD to achieve cholesterol efflux, although the detailed mechanism by which cholesterol regulates ERAD processing of NRF1 remains poorly understood [7]. Moreover, melatonin increases ERAD gene expression (*Hrd1*, *Vcp*, and *Os9*), leading to the suppression of tunicamycin-induced ER stress as well as senescence [10]. These findings indicate that melatonin regulates ERAD-dependent NRF1 translocation, which mediates the efflux of cholesterol present at high levels.

Clinical reports indicate that the wound healing process in some patients with obesity is extremely slow, contributing to a higher risk of infection and diminished quality of life [11]. Furthermore, metabolic diseases such as obesity increase the production of reactive oxygen species (ROS), and consequently, induce a delay in vasculogenesis, granulation, and re-epithelialization of wound sites [12]. To manipulate obesity-related wounds, multiple promising therapies using stem cells are currently under investigation. Umbilical cord blood-derived mesenchymal stem cells (UCB-MSCs) have low immunity and immunomodulatory effects, thereby increasing the survival of transplanted cells and decreasing the risk of graft-versus-host disease [13]. Furthermore, UCB-MSCs are widely used to accelerate tissue regeneration because of their ability to self-renew, mediate the paracrine effects of immunomodulatory and vasculogenic cytokines, and differentiate into diverse cell lineages [14]. However, the therapeutic efficacy of stem cell transplantation for the treatment of patients with obesity accompanied by hyperlipidemia and hypercholesterolemia is lower than that achieved for treating patients without obese [15, 16]. High levels of circulating cholesterol increase its intracellular levels, and excess cholesterol induces ER stress, production of NADPH oxidase-dependent ROS, and oxidative stress of sufficient intensity to cause cell death [17]. Furthermore, the cholesterol transporter is closely associated with the levels of intracellular cholesterol and ROS as well as apoptosis when cells are exposed to high cholesterol concentrations [18]. Considering that excessive levels of intracellular cholesterol increases cell death and that melatonin regulates cholesterol levels, examining the cholesterol regulatory mechanism of melatonin can suggest the possibility of improved therapeutic effects of UCB-MSCs for treating skin wound healing in patients with obesity. For this purpose, we investigated UCB-MSCs in vivo and in vitro to identify the regulatory mechanism and effects of melatonin on intracellular cholesterol levels and cholesterol-induced apoptosis.

## Methods

### Materials

UCB-MSCs were acquired from Kang Stem Biotech (Seoul, Korea). Fetal bovine serum (FBS) and antibiotics were purchased from Hyclone (Logan, UT, USA) and Gibco (Grand Island, NY, USA), respectively. 4-P-PDOT (#SML1189), cholesterol (#C4951), DIDS (#D3514), melatonin (#M5250), and Ver155008 (#SML0271) were purchased from Sigma-Aldrich (St. Louis, MO, USA). Mithramycin A (#1489) was purchased from Tocris (Minneapolis, MN, USA). Antibodies against ABCA1 (#ab18180), α-SMA (#ab5694), and Sp1 (#ab227383) were purchased from Abcam (Cambridge, England) and against HSF1 (#H00003297-A01) were purchased from Abnova (Taipei, Taiwan). Antibodies against NRF1 (#8052S) and cleaved-caspase 3 (#9661) were purchased from Cell Signaling Technology (Beverly, MA, USA), and those against BiP (#MA5-27686) and phospho-Sp1 (Thr453, #PA5-38333) were purchased from Invitrogen (Carlsbad, CA, USA). Antibodies against MT1 (#NBP1-71113) were purchased from Novus Biologicals (Littleton, CO, USA). Antibodies against β-actin (#sc-47778), caspase 9 (#sc-8355), lamin A/C (#sc-20681), and MT2 (#sc-28453) were purchased from Santa Cruz Biotechnology (Dallas, TX, USA). All primers for mRNA and miRNA were purchased from Cosmogenetech (Seoul, Korea). Small interfering RNAs (siRNAs) were purchased from Bioneer (Deajeon, Korea).

### Cell culture

UCB-MSCs were cultured with α-minimum essential medium (α-MEM; Hyclone, #SH30265.01) containing 10% FBS and 1% antibiotics at 37 °C in an atmosphere containing 5% CO_2_. Cells cultured to 80% confluency were incubated with serum-free α-MEM for 24 h before treatment with reagents.

### Cholesterol quantification assay

Cells were harvested, and cholesterol was extracted with 200 μL of chloroform:isopropanol:NP40 (7:11:0.1) solution. After spin down at maximum speed to remove debris, the supernatant was transferred to new tubes and dried at 50 °C for 4 h. The pellets were resolved in assay buffer and incubated with enzyme-mixed assay buffer at 37 °C for 30 min. The absorbance at 570 nm was measured using a microplate spectrophotometer (Epoch 2™; BioTek, Winooski, VT, USA).

### Western blot analysis

The collected cells were lysed with RIPA lysis buffer (Atto, Tokyo, Japan, #AE6500). Samples were prepared using sample buffer, loaded into 8–12% sodium dodecyl sulfate–polyacrylamide gels, and transferred to polyvinylidene fluoride membranes. The membranes were washed with tris-buffered saline containing 0.2% Tween-20 (TBST) (10 min, three times) and blocked with 5% skim milk in TBST at room temperature (RT) for 30 min. After incubation with a primary antibody solution in TBST (1:2000 dilution) at 4 °C overnight, the membranes were incubated with anti-mouse or rabbit horseradish peroxidase-conjugated secondary antibody solution at RT for 2 h. The β-actin band was used as a loading control. Collected images of bands were analyzed using ImageJ software.

### Immunocytochemistry analysis

UCB-MSCs were cultured in confocal dishes (SPL, Pocheon, Korea, #200350), fixed with 70% acetone in PBS for 10 min, and then permeabilized with 0.1% TritonX-100 in PBS for 10 min. After blocking with 5% normal goat serum (NGS) in PBS solution for 30 min, cells were incubated with primary antibodies in 5% NGS solution (1:100 dilution) at 4 °C overnight and with secondary antibodies in 5% NGS solution (1:200 dilution) at RT for 2 h. The cells were visualized using a super-resolution radial fluctuation imaging system (Andor Technology, UK). The relative fluorescence intensities of nuclear NRF1 and total NRF1 or co-localization of NRF1 and BiP were quantified using Fiji software.

### Transfection of siRNAs or microRNA mimics

For transfection, *NRF1* siRNAs or microRNA-597 (miR-597) mimics (25 nM) were incubated with TurboFect™ transfection reagent (Thermo Fisher, #R0531) and transfected to UCB-MSCs for 24 h. The sequences of the siRNAs and microRNA mimics used are presented in Supplementary Table 1.

### Quantitative polymerase chain reaction for analyzing mRNA and microRNA expressions

Total RNA samples were extracted using RNA extraction kits (Takara, Otsu, Shiga, Japan, #9767). Then, 1 μg of RNA was reverse-transcribed into cDNA using reverse transcription-PCR premix (iNtRON Biotechnology, Seongnam, Korea, #25081). The cDNA samples of mRNA were amplified using TB™ Green Premix (Takara, #RR420A) and primers for the target RNAs. The relative expression levels of mRNAs and microRNAs were quantified using 2^−^^ΔΔCt^ analysis. The primer sequences are presented in Supplementary Table 2.

### MicroRNA microarray

Biotinylated RNA strands were hybridized at 48 °C for 18 h on an Affymetrix GeneChip miRNA 4.0 Array (Affymetrix, Santa Clara, CA, US). The arrays were analyzed using an Affymetrix GeneChip scanner with associated software. The miRNA expression levels were calculated using Transcriptome Analysis Console. Relative signal intensities were generated for each miRNA using the Robust Multi Array Average algorithm. The microarray data were analyzed using a row *z*-score.

### Annexin V-FITC/PI staining

UCB-MSCs (1 × 10^5^ cells) were detached from the culture dishes, and collected cells were suspended in binding buffer. Annexin V-FITC and PI (BD Bioscience, Franklin Lakes, NJ, USA, #556547) were added to the samples, and the samples were incubated for 15 min at RT. The apoptotic cells were measured and analyzed using flow cytometry (CytoFlex; Beckman Coulter, Fullerton, CA, USA). Annexin V-FITC-positive and PI-positive (Q2) and Annexin V-FITC-positive and PI-negative (Q4) cells were considered to have undergone apoptosis.

### Water-soluble tetrazolium salt (WST-1) cell viability assay

After washing with PBS, cells were incubated in 10% EZ-Cytox™ (Daeil Lab service, Korea, #EZ-1000) solution in 100 μL of medium at 37 °C for 30 min. The absorbance at 450 nm was then measured using a microplate spectrophotometer (Epoch 2™, BioTek).

### Trypan blue exclusion assay

UCB-MSCs were washed with PBS, detached, and spun down at 3000 rpm. The cell pellet was suspended with 0.4% trypan blue (Sigma–Aldrich, #T6146) in PBS to stain dead cells. The number of trypan blue-stained and blue-unstained cells was counted using a Countess II FL Automated Cell Counter (Thermo Fisher).

### Measurements of intracellular ROS

The cells were washed with PBS and incubated in 1 mM CM-H_2_DCFDA (Thermo Fisher, #C6821) in culture media at 37 °C for 30 min. The fluorescence intensity of CM-H_2_DCFDA was measured using a luminometer at excitation and emission wavelengths of 485 and 535 nm.

### Mouse skin wound healing model

A high-fat diet (HFD, 60 kcal% fat, Research Diets Inc., NJ, USA) was provided to 6-week-old male Institute of Cancer Research (ICR) mice for 14 weeks. All mice were anesthetized with a mixture of Alfaxan™ (80 mg/kg, Jurox Pty Ltd., Rutheford, Australia) and xylazine HCl (10 mg/kg, Rompun™, Bayer, Leverkusen, Germany). The backs of anesthetized mice were shaved and scrubbed with organic iodine solution and 70% ethanol solution for disinfection during surgery. A wound was made in the back skin using a 6-mm-diameter circular biopsy punch. Then, 1 × 10^6^ UCB-MSCs were injected intradermally at three sites around each wound. Intraperitoneal injection of melatonin was conducted (30 mg/kg/day) for 12 days after wound formation. All mice were sacrificed at post-injection day 12. A serum biochemistry instrument was used to measure total cholesterol levels. The skin samples were fixed with 4% paraformaldehyde (PFA) and dehydrated with 20% and 30% sucrose solutions. Dehydrated skin samples were embedded in optimum cutting temperature compound (Sakura Finetek, CA, USA, #4583) and stored at − 80 °C. Frozen skin samples were cut into 20-μm-thick sections and mounted on silane-coated slides.

### Hematoxylin and eosin (H&E) staining

Skin tissue samples were mounted on slides, fixed with 4% PFA for 5 min, and then stained with H&E for 5 min. Samples were washed with 70%, 95%, and 100% ethanol three times and then incubated in xylene for 5 min. All images were acquired using the Eclipse Ts2™ fluorescence microscope (Nikon, Tokyo, Japan). Histological evaluations were performed blindly.

### Immunohistochemistry

Skin samples on slides were fixed in 80% acetone solution for 20 min. Slides were washed in PBS and incubated in 5% NGS for 30 min. Samples were incubated with the primary antibody in PBS containing 0.2% Tween-20 (PBST) for 2 h. After incubating with secondary antibodies in PBST (1:100 dilution) for 1 h, images were acquired using the Eclipse Ts2™ fluorescence microscope.

### Statistical analysis

Statistical analysis and graphing were performed using GraphPad Prism version 6.0 (GraphPad Inc., San Diego, CA, USA) statistical software. The mean values of treatment groups were compared with those of the control group using the Student’s *t* test, and differences among three or more experimental groups were analyzed using one-way analysis of variance with Dunnett’s multiple comparison test. *p* < 0.05 was considered statistically significant.

## Results

### Effect of melatonin on ABCA1-induced cholesterol efflux in the presence of high cholesterol concentrations

**Fig. 1:**
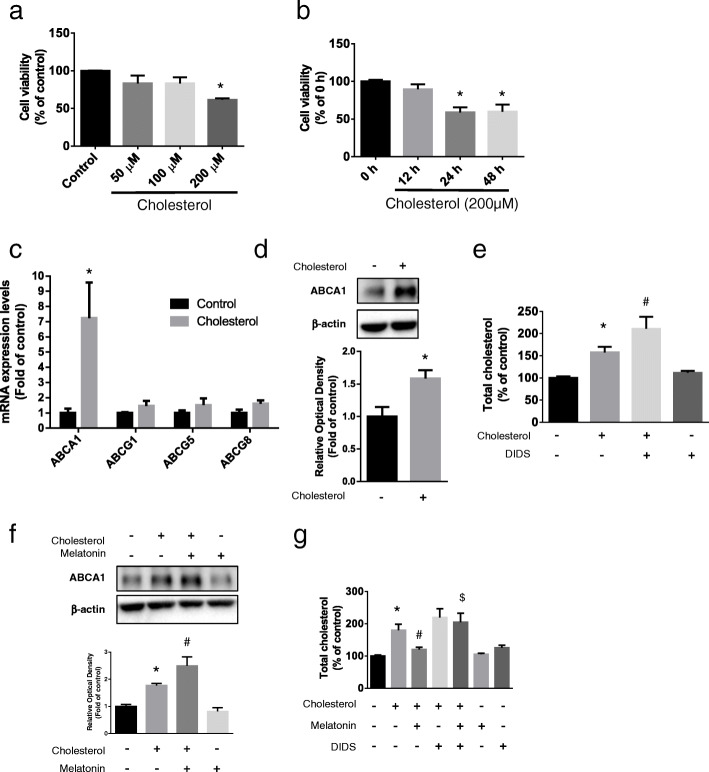
Effects of melatonin on ABCA1 expression and intracellular cholesterol level under high cholesterol condition. **a**, **b** UCB-MSCs were treated with various concentrations and time and then viability was measured by trypan blue exclusion assay (*n* = 5). **c**, **d** UCB-MSCs were treated with 200 μM cholesterol for 24 h. **c** The expression levels of cholesterol transporter genes (*ABCA1*, *ABCG1*, *ABCG5*, and *ABCG8*) were measured by qPCR (*n* = 5). **d** The expression level of ABCA1 was quantified by western blot assay and relative optical density was measured by ImageJ (*n* = 5). **e** UCB-MSCs were pretreated with DIDS (10 μM) for 30 min prior to cholesterol treatment (200 μM, 24 h) and cellular cholesterol level was measured by cholesterol quantification kit (*n* = 5). **f** Melatonin (1 μM) was pretreated for 30 min before cholesterol treatment (200 μM, 24 h) and the expression level of ABCA1 was measured by western blotting (*n* = 5). **g** UCB-MSCs were pretreated with DIDS or melatonin prior to cholesterol treatment and their total cholesterol levels were measured by quantification kit (*n* = 5). All blot images are representative and quantitative data are presented as a mean ± standard error of the mean. *p < 0.05 vs control, ^#^*p* < 0.05 vs cholesterol, ^$^*p* < 0.05 vs melatonin + cholesterol

On measuring apoptosis of UCB-MSCs treated with different concentrations of cholesterol (0–200 μM), we found that 200 μM cholesterol significantly reduced the survival of UCB-MSCs after 24 h (Fig. 1a, b). Cholesterol efflux depends on a membrane transporter and, therefore, we determined changes in the expression levels of genes encoding cholesterol transporters. The *ABCA1* mRNA level was significantly increased in UCB-MSCs (Fig. 1c). Western blot results showed that the ABCA1 protein level also increased (Fig. 1d). We next used the ABCA1 inhibitor DIDS (10 μM) to determine the role of ABCA1 in the regulation of intracellular cholesterol levels. DIDS treatment followed by the addition of cholesterol significantly increased the levels of intracellular cholesterol compared with cholesterol treatment alone (Fig. 1e). Western blot results showed that melatonin and cholesterol treatment further increased ABCA1 expression compared with cholesterol treatment alone (Fig. 1f). Cells were pretreated with DIDS or melatonin, and the intracellular cholesterol level was measured to determine the relationship between melatonin-induced ABCA1 expression and the regulation of intracellular cholesterol levels. High concentrations of cholesterol added to the cultures increased the intracellular cholesterol levels, which were decreased when the cells were pretreated with melatonin. However, combined treatment with melatonin and DIDS did not significantly increase cholesterol levels when cells were pretreated with DIDS. These results indicate that ABCA1 is required to regulate the action of melatonin on intracellular cholesterol levels (Fig. 1g). Collectively, the levels of ABCA1 were increased when cells were treated with high concentrations of cholesterol, and melatonin increased ABCA1 levels and cholesterol efflux.

### Effects of melatonin on high cholesterol-induced intracellular ROS levels and apoptosis

**Fig. 2:**
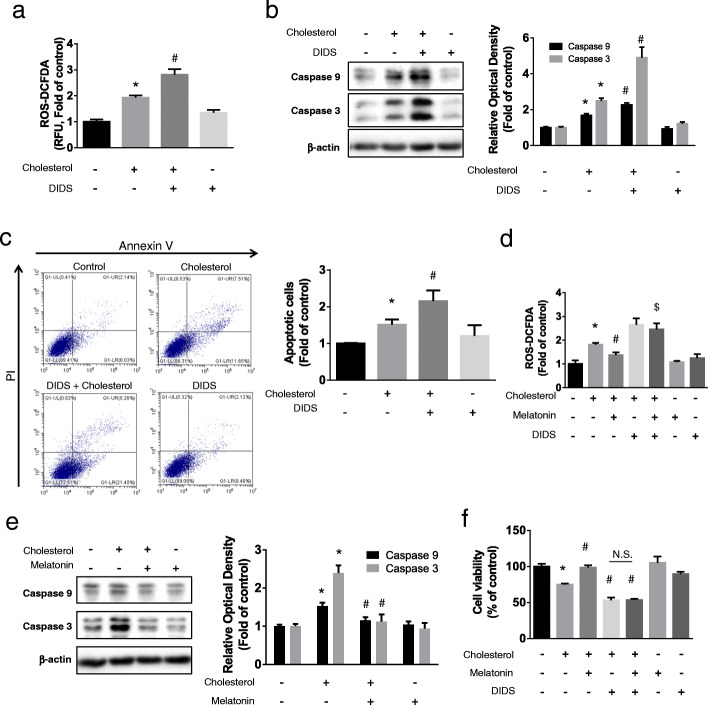
Effects of melatonin on high cholesterol-induced ROS accumulation and apoptosis. **a**–**c** UCB-MSCs were pretreated with DIDS (10 μM) prior to cholesterol treatment (200 μM, 24 h). **a** The intracellular ROS level was measured by CM-H_2_DCFDA. The relative fluorescence units (RFU) was measured by luminometer (*n* = 5). **b** The expression level of caspase 9 and 3 were quantified by western blotting (*n* = 5). **c** The percentage of apoptosis was analyzed with Annexin V/PI assay. Annexin V-FITC-positive and PI-positive (Q2), and Annexin V-FITC-positive and PI-negative (Q4) UCB-MSCs were considered as apoptotic (*n* = 5). **d** The cells were pretreated with melatonin (1 μM) or DIDS (10 μM) prior to cholesterol treatment (200 μM, 24 h), and the level of total ROS was measured by CM-H_2_DCFDA (*n* = 5). **e** The cells were pretreated with melatonin (1 μM) prior to cholesterol treatment (200 μM, 24 h) and then the expression levels of caspase 9 and 3 were measured by western blotting (*n* = 5). **f** The cells were pretreated with melatonin (1 μM) or DIDS (10 μM) prior to cholesterol treatment (200 μM, 24 h), and cell viability was measured with WST-1 assay (*n* = 5). All blot images are representative and quantitative data are presented as a mean ± standard error of the mean. *p < 0.05 vs control, ^#^*p* < 0.05 vs cholesterol, ^$^*p* < 0.05 vs melatonin + cholesterol, n.s. means not significant

To evaluate the correlation between high cholesterol levels and apoptosis, we measured cholesterol-induced ROS. Cholesterol treatment significantly increased ROS levels, which were further increased when cells were pretreated with DIDS (Fig. 2a). The effects of cholesterol on apoptotic cell death were assessed using western blot and Annexin V/PI assays. The expression levels of caspase 9 and caspase 3 and the percentages of cell death were significantly increased following cholesterol treatment, and DIDS treatment further increased the level of apoptosis of cholesterol-treated cells (Fig. 2b, c). Cells were treated with cholesterol combined with DIDS or melatonin to determine the effect of melatonin on cholesterol-induced ROS levels and the involvement of ABCA1-dependent regulation of cholesterol efflux. Melatonin significantly decreased cholesterol-induced ROS, and combined treatment with DIDS and melatonin did not significantly change ROS levels compared with that in cells pretreated with DIDS (Fig. 2d). Western blot analysis showed that melatonin decreased cholesterol-induced caspase 9 and caspase 3 expression levels (Fig. 2e). WST-1 assays of cell viability showed that melatonin restored the viability of cholesterol-treated cells to the control level. There was no significant difference between treatment with DIDS before exposure to cholesterol and treatment with cholesterol combined with DIDS and melatonin (Fig. 2f). Thus, melatonin alleviated the effects of cholesterol-induced ROS and apoptosis through cholesterol efflux mediated by increased ABCA1 expression.

### Effect of melatonin on the therapeutic efficacy of transplanted UCB-MSCs in a mouse model of HFD-induced obesity

**Fig. 3:**
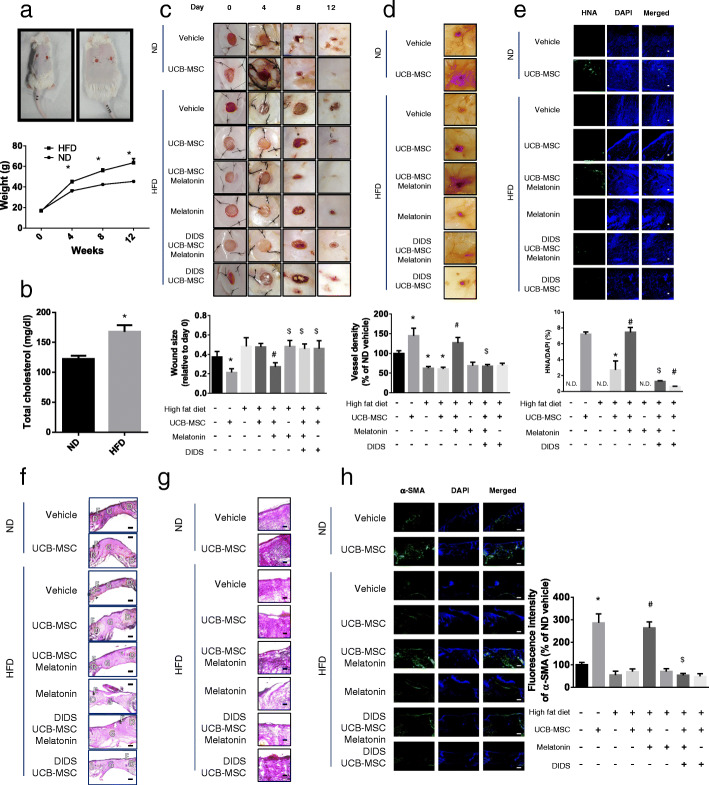
Effects of melatonin on transplanted cells in the HFD-induced obese mouse model. Obese mouse model was prepared by feeding high-fat-diet (HFD) for 14 weeks. **a**, **b** Weight and the level of total cholesterol in serum of HFD-induced obese mice were compared with normal diet (ND) feeding mice (*n* = 7, *p < 0.05 vs ND). Mice were divided into the 8 groups and skin wound healing model was conducted. After making wounds on the skin of the back, UCB-MSCs with or without DIDS pretreatment were inoculated near the wound site and melatonin (30 mg/kg/day) was injected to the mice of melatonin treatment group. **c**, **d** Wound size and vasculogenesis around the wound site were observed (*n* = 7, *p < 0.05 vs ND + vehicle, ^#^*p* < 0.05 vs HFD + UCB-MSC, ^$^*p* < 0.05 vs HFD + UCB-MSC + melatonin). **e** Immunohistochemistry (IHC) was conducted with an antibody for human nuclear antigen (HNA, green) and DAPI (blue) staining and visualized by fluorescence microscopy. Scale bars were set as 100 μm (magnification × 100). The percentage of HNA-positive cells in total cells were analyzed with Fiji software (*n* = 5, *p < 0.05 vs ND + UCB-MSC, #*p* < 0.05 vs HFD + UCB-MSC, ^$^*p* < 0.05 vs HFD + UCB-MSC + melatonin, N.D. indicates not detected). **f** H&E staining was conducted on tissues of wound site and compared. Scale bars are 200 μm (magnification × 50). D dermis, E epidermis, G granulation tissue. **g** Masson’s trichrome staining was conducted on skin wound tissues. Scale bars are 100 μm (magnification × 100) **h** IHC was conducted with an antibody for alpha-smooth muscle antigen (α-SMA, green) and DAPI (blue) staining. Scale bars were set as 200 μm (magnification × 50), (*n* = 5, *p < 0.05 vs ND + UCB-MSC, ^*#*^*p* < 0.05 vs HFD + UCB-MSC, ^$^*p* < 0.05 vs HFD + UCB-MSC + melatonin). All images are representative and quantitative data are presented as a mean ± standard error of the mean

Obesity was induced in mice by feeding them HFD for 14 weeks to determine the effect of melatonin on UCB-MSCs transplanted into skin wounds. The mice had higher weight and total cholesterol levels than mice fed normal diet (ND) (Fig. 3a, b). To determine the difference between the efficacy of UCB-MSC transplantation into obese and normal mice, we used a skin wound healing model. On day 12 after causing the skin injury, the transplantation efficacy was compared by measuring the sizes of wound closures. The wound sizes of UCB-MSC-transplanted HFD mice were significantly larger than that of UCB-MSC-transplanted ND mice. Melatonin restored the wound sizes in UCB-MSC-transplanted HFD mice to those in UCB-MSC-transplanted ND mice. However, in mice transplanted with DIDS-pretreated UCB-MSC cells, melatonin did not show the enhancing wound healing effect of UCB-MSC (Fig. 3c). The effect of UCB-MSCs on vasculogenesis was recovered by melatonin treatment, which was decreased in HFD mice (Fig. 3d). To determine the antiapoptotic effect of melatonin on engrafted UCB-MSCs, we measured human nuclear antigen expression in wounded tissues. Melatonin-treated HFD mice harbored the same number of UCB-MSCs as transplanted ND mice, which decreased when the cells were pretreated with DIDS (Fig. 3e). Histological scores for re-epithelialization indicated that melatonin treatment increased the extent of re-epithelialization in UCB-MSC-transplanted mice compared with that in other HFD mice (Fig. 3f). Furthermore, we measured the level of fibrosis to evaluate the efficacy of wound healing therapy with Masson trichrome staining and immunohistochemistry using α-SMA antibody. The results showed that fibrosis in granulation tissues increased with melatonin treatment, whereas inhibition of ABCA1 in UCB-MSCs reduced these effects (Fig. 3g and h). Thus, the efficacy of MSC transplantation for mice with obesity was improved by the ABCA1-dependent effects of melatonin.

### Role of MT2-dependent BiP/NRF1 inhibition of melatonin-induced ABCA1 expression

**Fig. 4:**
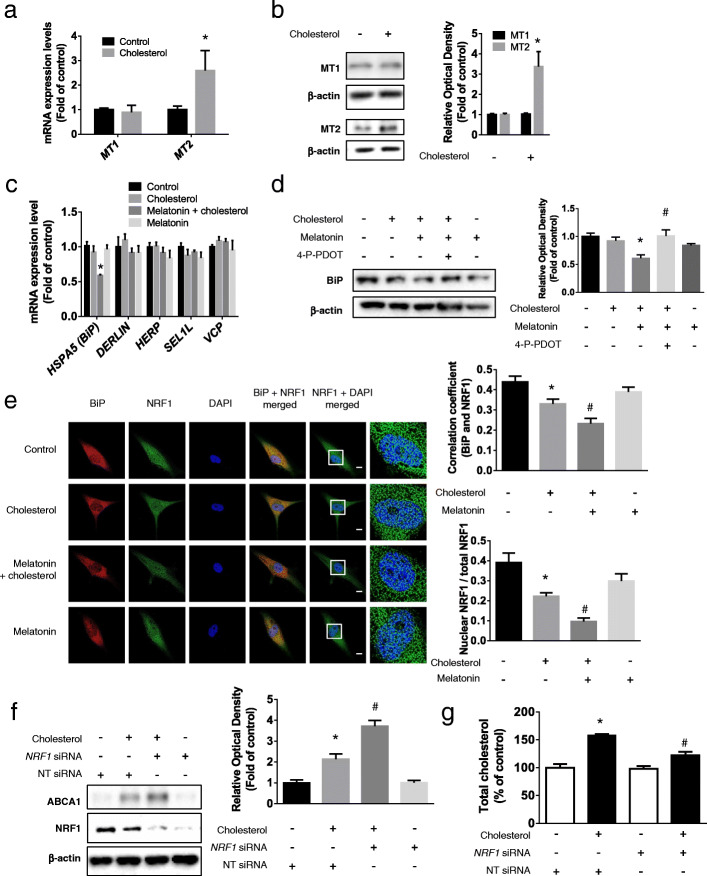
Involvement of MT2/Bip/NRF1 pathway in melatonin-induced ABCA1 expression. **a** and **b** Cholesterol (200 μM, 24 h) was treated to UCB-MSCs and the changes in expression levels of MT1 and MT2 were measured by qPCR or western blotting (*n* = 5, *p < 0.05 vs control). **c** Melatonin (1 μM) was pretreated to UCB-MSCs before cholesterol treatment (200 μM, 24 h) and then the expression levels of *HSPA5*, *DERLIN*, *HERP*, *SEL1L*, and *VCP* were compared by qPCR (*n* = 5, *p < 0.05 vs cholesterol). **d** MT2 inhibitor, 4-P-PDOT (10 μM) were pretreated prior to melatonin treatment (1 μM) and the expression level of BiP was measured by western blotting (*n* = 5, *p < 0.05 vs control, ^#^*p* < 0.05 vs cholesterol + melatonin). **e** Immunocytochemistry was conducted with NRF1 (green) and BiP (red) specific antibodies and DAPI (blue) and then visualized by SRRF imaging system. Scale bar was set as 8 μm (magnification × 1000) (*n* = 5, *p < 0.05 vs control, ^#^*p* < 0.05 vs cholesterol). **f**, **g** *NRF1* or NT siRNA was transfected to UCB-MSCs using transfection reagents prior to cholesterol treatment (200 μM, 24 h). The expression levels of ABCA1 and NRF1 were measured by western blotting and the level of total cholesterol was quantified with cholesterol quantification kit (*n* = 5, *p < 0.05 vs NT siRNA, ^#^*p* < 0.05 vs NT siRNA + cholesterol). All blot and images are representative and quantitative data are presented as a mean ± standard error of the mean

We determined the levels of mRNAs and proteins of melatonin receptors 1A and 1B using qPCR and western blotting; the *MT2* mRNA and protein expression level were increased in cholesterol-treated UCB-MSCs (Fig. 4a, b). NRF1 regulates cholesterol homeostasis by repressing the activity of LXRα, which is a major transcription factor that drives the expression of ABCA1. Furthermore, ERAD regulates the nuclear translocation of NRF1. Therefore, we determined the melatonin-dependent changes in the mRNA expression of ERAD-related proteins under conditions of high cholesterol concentrations. qPCR results show that cholesterol treatment following melatonin treatment significantly decreased the levels of *HSPA5* mRNA (Fig. 4c). We next used the competitive MT2 antagonist 4-phenyl-2-propionamidotetralin (4-P-PDOT) to determine if the regulatory effect of melatonin on BiP was MT2-dependent. The western blot results show that 4-P-PDOT treatment abolished the melatonin-induced decrease of BiP expression (Fig. 4d). We performed immunocytochemistry to confirm these findings. The results showed that melatonin treatment further decreased the co-localization of BiP and NRF1 and the nuclear translocation of NRF1 compared with that in cholesterol-treated cells (Fig. 4e). To confirm the regulatory effect of NRF1 on ABCA1 expression and cholesterol efflux, we determined the effect of an *NRF1*-specific siRNA on ABCA1 levels and intracellular cholesterol levels in the presence of high cholesterol concentrations. Under these conditions, *NRF1* knockdown increased ABCA1 levels and decreased intracellular cholesterol levels compared with those in cells transfected with NT siRNA (Fig. 4f, g). Thus, melatonin increased ABCA1 expression via MT2-dependent inhibition of BiP expression and nuclear translocation of NRF1.

### Role of BiP in the nuclear translocation of NRF1 and ABCA1 expression in the presence of high cholesterol concentrations

**Fig. 5:**
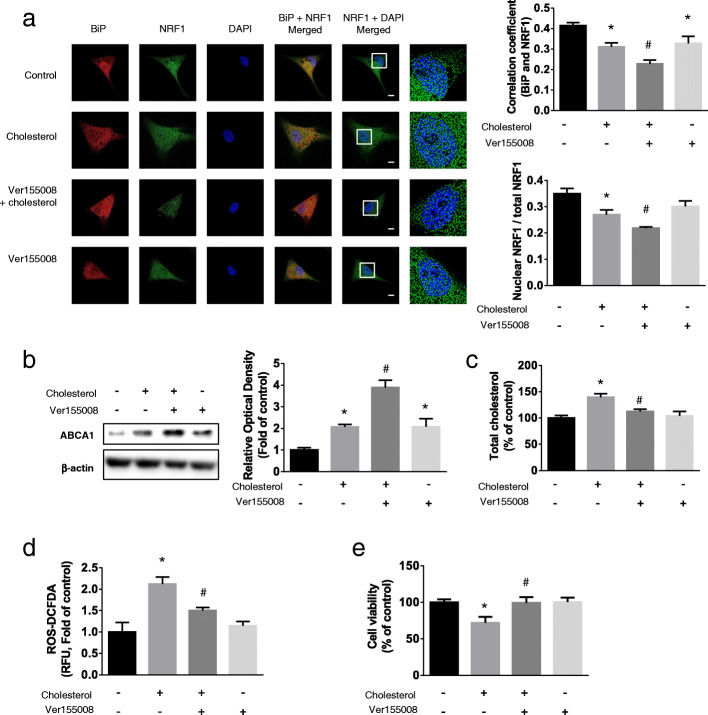
Role of BiP in nuclear NRF1 and ABCA1 expression under high cholesterol condition. **a**–**e** UCB-MSCs were pretreated BiP inhibitor, Ver155008 (10 μM) prior to cholesterol treatment (200 μM, 24 h). **a** Immunocytochemistry was conducted with NRF1 (green), BiP (red) specific antibodies and DAPI (blue), and then visualized by SRRF imaging system. Scale bar was set as 8 μm (magnification × 1000). The co-localization of BiP and NRF1, nuclear translocation of NRF1 were analyzed with Fiji software. (*n* = 7). **b** The expression level of ABCA1 was quantified with western blotting (*n* = 5). **c** The concentration of the cellular cholesterol was measured with cholesterol quantification kit (*n* = 5). **d** Cells were incubated with CM-H_2_DCFDA and relative fluorescence units (RFU) were measured by luminometer (*n* = 5). **e** Cell viability was measured by trypan blue exclusion assay (*n* = 5). All blot and images are representative and quantitative data are presented as a mean ± standard error of the mean. *p < 0.05 vs control, ^#^*p* < 0.05 vs cholesterol

To determine the role of BiP in ERAD-dependent nuclear translocation of NRF1, we treated cells with the BiP inhibitor Ver155008 (10 μM) and performed immunocytochemistry to detect NRF1 and BiP co-localization as well as NRF1 nuclear translocation. Ver155008 decreased NRF1 and BiP co-localization to a greater extent than that in cholesterol-treated cells, and NRF1 nuclear translocation similarly decreased (Fig. 5a). We next determined whether decreased NRF1 nuclear translocation caused by BiP inhibition affected ABCA1 expression. The increase of ABCA1 levels in UCB-MSCs treated with Ver155008 and then with cholesterol was higher than that in cells treated with cholesterol alone (Fig. 5b). To evaluate the effect of BiP inhibition on NRF1-dependent regulation of the levels of intracellular cholesterol and ROS and cholesterol-induced apoptosis, we quantified cholesterol and ROS levels and used Annexin V/PI analyses to measure the level of apoptosis. We found that Ver155008 treatment followed by cholesterol treatment decreased intracellular cholesterol levels, ROS levels, and apoptosis of UCB-MSCs in the presence of high cholesterol concentrations (Fig. 5c, d, and e). Collectively, suppression of BiP decreased NRF1 nuclear translocation, which increased the levels of ABCA1 and decreased the levels of cholesterol as well as high-cholesterol-induced apoptosis.

### Role of Sp1-induced miR-597 in the inhibition of BiP expression by melatonin

**Fig. 6:**
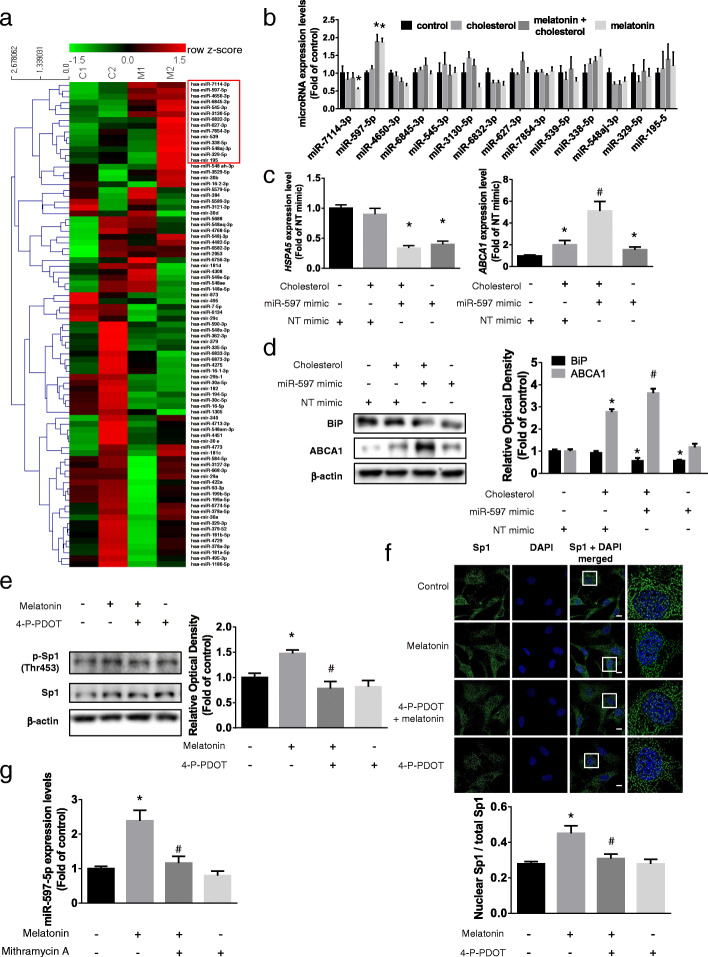
Involvement of melatonin-activated Sp1/miR-597 pathway in BiP suppression. **a** UCB-MSCs were treated with melatonin (1 μM, 24 h) and total RNAs were extracted. MicroRNA microarray was conducted and the expression levels of microRNAs were analyzed using hierarchical clustering with heatmap. **b** A cluster of expression-increased microRNAs was selected and their expression levels under high cholesterol and melatonin condition were analyzed by qPCR (*n* = 5, *p < 0.05 vs control). **c**, **d** miR-597 mimic was transfected to UCB-MSCs for 24 h prior to cholesterol treatment (200 μM, 12 h). The mRNA expressions of *HSPA5* and *ABCA1* and protein expression levels of BiP and ABCA1 were quantified with qPCR and western blotting (*n* = 5, *p < 0.05 vs NT mimic, #*p* < 0.05 vs NT mimic + cholesterol). **e**, **f** MT2 inhibitor 4-P-PDOT (10 μM) was pretreated prior to melatonin treatment (1 μM, 12 h). **e** The phosphorylation of Sp1 (Thr453) were measured by western blotting (*n* = 5, *p < 0.05 vs control, ^#^*p* < 0.05 vs melatonin). **f** Immunocytochemistry was conducted with Sp1 (green) specific antibody and DAPI (blue). Scale bar was set as 8 μm (magnification × 1000, *n* = 5, *p < 0.05 vs control, ^#^*p* < 0.05 vs melatonin). **g** Sp1 inhibitor Mithramycin A (5 nM) was pretreated prior to melatonin treatment (1 μM, 12 h), and the change of expression level of miR-597-5p was assessed through qPCR (*n* = 5, *p < 0.05 vs control, #*p* < 0.05 vs melatonin). All images are representative and quantitative data are presented as a mean ± standard error of the mean

We investigated the regulation of BiP expression by melatonin via the MT2 signaling pathway. For this, we determined if BiP expression was regulated by microRNAs. Eighty-three BiP-targeting microRNAs were selected from three databases (Genecards, microRNA.org, and Diana tools), and we performed microRNA microarray to select the microRNA candidates that increased following melatonin treatment. The microarray data were analyzed using row *z*-score with hierarchical clustering to compare the changes in expression levels of microRNAs, and we identified a cluster of 14 candidate microRNAs whose expression levels were increased following melatonin treatment (Fig. 6a). We conducted qPCR to confirm the expression levels of candidate microRNAs under cholesterol and melatonin conditions, and the results showed that miR-597-5p expression was significantly increased following melatonin treatment (Fig. 6b). We next used a microRNA mimic to determine whether miR-597-5p regulated BiP expression. The qPCR and western blot results revealed that miR-597-5p mimic suppressed BiP expression and upregulated ABCA1 expression compared with that in cholesterol-treated cells (Fig. 6c, d). Among transcription factors regulated by MT2-dependent signaling pathways and candidates that bind the miR-597 promoter region, we selected Sp1. First, we determined if the effect of melatonin on Sp1 activation was dependent on MT2. Thr453 phosphorylation of Sp1, which activates Sp1, increased with melatonin treatment but decreased when cells were pretreated with 4-P-PDOT. Immunocytochemistry results revealed that Sp1 nuclear translocation increased in response to melatonin treatment, which was inhibited when cells were pretreated with 4-P-PDOT (Fig. 6e, f). Next, to verify the role of Sp1 in the regulation of miR-597 expression, we pretreated cells with mithramycin A to inhibit Sp1 activation. The results revealed that mithramycin A decreased melatonin-induced miR-597 expression (Fig. 6g). Thus, melatonin decreased the expression of BiP via an MT2-Sp1-dependent miR-597 regulatory pathway.

## Discussion

We demonstrated that melatonin protected UCB-MSCs from cholesterol-induced apoptosis through a BiP/NRF1-dependent intracellular cholesterol efflux mechanism. In the presence of high cholesterol concentrations, LXRα-dependent responses sense and adapt to increased cholesterol efflux through the increased expression of ABCA1 [19]. We showed that ABCA1 was required for the downregulation of intracellular cholesterol levels and cholesterol-induced apoptosis. Furthermore, increased ABCA1 expression in the presence of high cholesterol concentrations did not protect the cells from cholesterol-induced apoptosis. A previous study showed that increasing the expression levels of cholesterol transporters upregulates cholesterol efflux, which is required for inhibiting cholesterol-induced apoptosis [20]. Another study demonstrated that ABCA1 overexpression suppresses the accumulation of cholesterol [21]. In the present study, melatonin treatment of UCB-MSCs reduced the levels of high-cholesterol-induced ROS and apoptosis and intracellular cholesterol levels by increasing ABCA1 expression. As shown here and in another study, DIDS combined with melatonin abolished cholesterol efflux mediated by ABCA1 without significantly changing the levels of ABCA1 or ROS [22]. The various effects of melatonin such as lowering oxidative stress, regulating lipid metabolism, and inducing receptor-mediated signal transduction could be involved in protecting UCB-MSC apoptosis under a high cholesterol environment [23–25]. Considering that intracellular cholesterol regulation is critical for oxidative stress, which activates numerous signaling pathways, including signal molecules related to oxygen and lipids, our proposed target can be of therapeutic importance. Because lipid-mediated oxidative stress could be increased in patients with obesity and HFD mice, oxidative stress is implicated in the pathogenesis, and it has been studied for its damaging effects [26]. We also found that cells pretreated with Ver155008 (BiP inhibitor) decreased intracellular cholesterol levels, ROS, and apoptosis of UCB-MSCs under high cholesterol conditions. These findings indicate that the protective effect of melatonin on high-cholesterol-induced ROS generation and apoptosis does not require ROS scavenging but rather inhibition of cholesterol accumulation. Therefore, we suggest that the protective effect conferred upon stem cells by melatonin is explained by the ABCA1-mediated removal of ROS generated in response to excess cholesterol levels.

Because of its occurrence and cost, skin wounds represent a primary health concern, and therapeutic approaches using UCB-MSCs have been studied to accelerate the wound healing process in preclinical and clinical studies [27]. However, many reports show that the effect of stem cell transplantation for promoting skin wound recovery was reduced in patients with obesity [15, 16]. In obesity, impairment of skin wound recovery can lead to tissue necrosis, which can diminish the patient’s quality of life and lead to complications [28]. Therefore, we conducted this study to improve the efficacy of UCB-MSCs transplantation therapy in patients with obesity, and the tissue regenerative effects were evaluated using the skin wound healing model. In a mouse model of obesity, using UCB-MSCs, we showed that melatonin treatment improved the efficacy of stem cell transplantation therapy, which was abolished following ABCA1 inhibition. Melatonin treatment did not significantly change the levels of plasma cholesterol in the mice, and analysis of the UCB-MSCs revealed a regulatory role that controls intracellular cholesterol levels. Previous investigations found that the administration of melatonin (10–30 mg/kg/day) for 4–8 weeks decreased total serum levels of cholesterol in sera harvested from HFD mice [29, 30]. In the present study, we injected melatonin (30 mg/kg/day) for 12 days after wound formation. Consistent with our results, two studies found that the administration of melatonin (4–10 mg/kg/day) for 2 weeks had no significant effect on serum cholesterol levels [31, 32]. These differences can be attributed to the duration of melatonin administration. When we focused on short-term treatment, melatonin affected the regulation of intracellular cholesterol levels in transplanted UCB-MSCs rather than cholesterol levels in the blood. Our study suggested that the decreased stem cell therapeutic efficacy in obese conditions was improved by an increase in survival rates of transplanted UCB-MSCs following melatonin treatment, although the plasma levels of cholesterol in obese mice were still high, which can lead to disease onset. Previous studies showed that melatonin treatment increases the efficacy of transplanted UCB-MSCs in skin wound healing experiments, whereas melatonin enhances immunoregulatory effects and the proliferation of adipose tissue-derived MSCs [2]. In contrast, we show here that inhibition of ABCA1 expression diminished the effects of melatonin on the engraftment rate of UCB-MSCs and skin wound healing, which indicates that the regulation of intracellular cholesterol levels by melatonin is important. A previous study found that the increased survival rate of transplanted UCB-MSCs was positively correlated with their regenerative potentials, such as wound regeneration, wound closure rate, and neovascularization [33]. Overall, the present study showed that UCB-MSC dysfunction during UCB-MSC transplantation into obese mice could be prevented by treatment with melatonin. Therefore, we proposed a melatonin co-treatment strategy to prevent a decrease in the efficacy of UCB-MSC transplantation therapy.

We demonstrated that melatonin decreased BiP expression through an MT2-dependent miR-597 regulatory pathway and that BiP was required for the nuclear translocation of NRF1. NRF1 reportedly regulates the antioxidant effect by increasing the transcription of antioxidant response element genes [34]. In contrast, under high cholesterol conditions, NRF1 regulates cholesterol homeostasis in the ER membrane by decreasing nuclear translocation and increasing ABCA1 expression [7]. Furthermore, our results showed that the decrease in high-cholesterol-induced ROS following melatonin treatment is ABCA1-dependent. Therefore, we investigated the role of NRF1 regulation by melatonin in ABCA1 expression under cholesterol conditions. Cholesterol treatment also increased the MT2 expression in UCB-MSCs, and another study found that ROS accumulation was significantly associated with MT2 expression [35]. We suggest, therefore, that high-cholesterol-induced ROS accumulation induces MT2 expression. If true, this process has important implications for the use of melatonin to control the levels of intracellular cholesterol because increased sensitivity to melatonin activates downstream pathways in the presence of high cholesterol levels. Evidence indicates that BiP contributes to ERAD by selecting substrates and assembly of the ERAD complex [36]. The ubiquitin-conjugating enzyme E2G2 together with the HRD1 complex polyubiquitinates substrates and the valosin-containing protein p97 to retro-transport substrates to the cytosol [37]. The expression levels and translocation of NRF1 are regulated by ERAD, suggesting the participation of BiP in the ERAD-dependent translocation of NRF1 [8]. Our results are consistent with this possibility because we show that BiP inhibition further decreased the cholesterol-induced reduction in NRF1 nuclear translocation, leading to increased ABCA1 expression. Some studies showed that melatonin treatment reduced stress-induced BiP expression [38, 39]. Our results show that melatonin treatment decreased the levels of *HSPA5* mRNA and BiP but did not change the expression and activation of its transcription factor. A study showed that melatonin regulates cellular physiological functions through microRNAs [40]. Further, microRNAs inhibit cancer cell proliferation and metastasis, and melatonin-induced miR-392a and miR-34b increase cancer cell apoptosis by inhibiting ABCB1/ABCB4 activity [41]. Moreover, melatonin regulates the transcription of microRNAs through the CEBP pathway or the Sp1 pathway [42]. We, therefore, focused on the regulatory effects of a melatonin-induced microRNA on *HSPA5* mRNA transcription. We demonstrated that melatonin treatment increased MT2-dependent phosphorylation and nuclear translocation of Sp1, which induced miR-597-5p expression to inhibit BiP. To the best of our knowledge, this is the first study to report that melatonin induces miR-597-5p expression. Further studies are required to define the mechanism by which miR-597 regulates BiP expression.

## Conclusions

We demonstrated that melatonin rescued UCB-MSCs from high-cholesterol-induced apoptosis by downregulating the expression of BiP as well as the nuclear translocation of NRF1, which plays an essential role in the regulation of intracellular cholesterol levels. This is the first report to reveal the novel role of melatonin in intracellular cholesterol efflux and the details of the underlying mechanism. We suggest focusing research efforts on developing a new strategy that employs melatonin to enhance the resistance of UCB-MSCs to high cholesterol levels to improve the efficacy of stem cell transplantation to treat patients with obesity.

## Supplementary Information

**Additional file 1: Supplementary Table 1.** Primer sequences for siRNA and micro RNA mimic. **Supplementary Table 2.** Primer sequences for mRNA and miRNA.

## Data Availability

The results and data sets used in this study are available from the corresponding author on reader’s request.

## Abbreviations

ABCA1: ATP-binding cassette subfamily A member 1
BiP: Binding immunoglobulin protein
NRF1: Nuclear factor erythroid 2-related factor 1
UCB-MSCs: Umbilical cord blood-derived mesenchymal stem cells
ERAD: ER-associated degradation
