# Supplementary material for: Melatonin activates ABCA1 via the BiP/NRF1 pathway to suppress high-cholesterol-induced apoptosis of mesenchymal stem cells
Source: Stem Cell Res Ther. 2021 Feb 5;12:114. doi: 10.1186/s13287-021-02181-4 (PMC7866631; doi:10.1186/s13287-021-02181-4)
Supplement: Supplementary file 1 — Additional file 1: Supplementary Table 1. Primer sequences for siRNA and micro RNA mimic. Supplementary Table 2. Primer sequences for mRNA and miRNA. [file 13287_2021_2181_MOESM1_ESM.docx]

**Title**

Melatonin activates ABCA1 via the BiP/NRF1 pathway to suppress high-cholesterol-induced apoptosis of mesenchymal stem cells

**Authors**

Jun Sung Kim^1,a^, Young Hyun Jung^1,a^, Hyun Jik Lee^2,3^, Chang Woo Chae^1^, Gee Euhn Choi^1^, Jae Ryong Lim^1^, Seo Yihl Kim^1^, Joo Eun Lee^1^, and Ho Jae Han^1, *^

**Affiliations**

^1^Department of Veterinary Physiology, College of Veterinary Medicine, Research Institute for Veterinary Science, and BK21 PLUS Program for Creative Veterinary Science Research, Seoul National University, Seoul, 08826, Republic of Korea*.*

^2^Laboratory of Veterinary Physiology, College of Veterinary Medicine, Chungbuk National University, Cheongju, Chungbuk, 28644, Republic of Korea

^3^Institute for Stem Cell & Regenerative Medicine (ISCRM), Chungbuk National University, Cheongju, Chungbuk, 28644, Republic of Korea

^a^These authors contributed equally to this work

^*^Corresponding author**:** Ho Jae Han, D.V. M, Ph.D

Professor of Department of Veterinary Physiology

College of Veterinary Medicine, Seoul National University, Seoul, 08826, Republic of Korea

Tel: +82-2-880-1261

E-mail address: [hjhan@snu.ac.kr](mailto:hjhan@snu.ac.kr)

Tel: +82-2-880-1261

Fax: +82-2-880-2732

Supplementary Table 1. Primer sequences for siRNA and micro RNA mimic.

|  | Sequence |
| --- | --- |
| NRF1 sense | GAGAACGGACGACCCUACU |
| NRF1 antisense | AGUAGGGUCGUCCGUUCUC |
| MT2 sense | GAGAACGGCUCCUUCGCCA |
| MT2 antisense | GGUAAUUUGUUCUUGGUGA |
| Non-targeting sense | UAGCGACUAAACACAUCAA |
| Non-targeting antisense | UUGAUGUGUUUAGUCGCUA |
| hsa-miR-597 mimic | UGUGUCACUCGAGACCACUGU |

Supplementary Table 2. Primer sequences for mRNA.

| Primer | Sequence |
| --- | --- |
| ABCA1-F | AACAGTTTGTGGCCCTTTTG |
| ABCA1-R | AGTTCCAGGCTGGGGTACTT |
| ABCG1-F | ACGCAGTTCTGCATCCTCTT |
| ABCG1-R | CGGAGTTGCTCAAGACCTTC |
| ABCG5-F | CTCCTACAGCGTCAGCCAC |
| ABCG5-R | CGTTCACATACACCTCCCCC |
| ABCG8-F | GGACCTGACCAGCATTGACA |
| ABCG8-R | GCATCTTCGTAGGACTCGGG |
| HSPA5(BiP)-F | CACTCCTGAAGGGGAACGTC |
| HSPA5(BiP)-R | TCAACCACCTTGAACGGCAA |
| DERLIN 1-F | TTCTTGCACACATGCCTCTC |
| DERLIN 1-R | GCTGAGAAAACGCTTCATCC |
| Sel1L-F | AAGCCCTGGAGAGAGTGTCA |
| Sel1L-R | CCCCAAGAGCTCCAAATGTA |
| HERP-F | ACTTGCTTCCAAAGCAGGAA |
| HERP-R | CCCTTTGCCTTAAACCATCA |
| VCP-F | ATCCGTGAATCCATCGAGAG |
| VCP-R | GACTCTGCTGAAGGGTCTGG |
| MT1-F | GATCCTGGTTCTCCAGGTCA |
| MT1-R | CATTGAGGCAGCTGTTGAAA |
| MT2-F | CGGAACGCAGGTAATTTGTT |
| MT2-R | TAATGGCGATGGCAGTGATA |
| hsa-miR-7114-3p | GTGACCCACCCCTCTC |
| hsa-miR-195-5p | CGCAGTAGCAGCACAGA |
| hsa-miR-329-5p | GCAGGAGGTTTTCTGGGT |
| hsa-miR-338-5p | GCAGAACAATATCCTGGTGCT |
| hsa-miR-539-5p | GCAGGGAGAAATTATCCTTGGT |
| hsa-miR-545-3p | CGCAGTCAGCAAACATTTATTG |
| hsa-miR-548aj-3p | AGCGCAGTAAAAACTGCAA |
| hsa-miR-597-5p | GTGTCACTCGATGACCAC |
| hsa-miR-627-3p | CGCAGTCTTTTCTTTGAGACTC |
| hsa-miR-3130-5p | CCAGTCTCCGGTGCAG |
| hsa-miR-4650-3p | GCGCAGAGGTAGAATGAG |
| hsa-miR-6832-3p | CGCAGACCCTTTTTCTCT |
| hsa-miR-6845-3p | AGCCTCTCCTCCCTGT |
| hsa-miR-7854-3p | GTGAGGTGACCGCAGA |
| Universal primer | Qiagen miScript Universal Primer |
